# Delamination Strength and Elastin Interlaminar Fibers Decrease with the Development of Aortic Dissection in Model Rats

**DOI:** 10.3390/bioengineering10111292

**Published:** 2023-11-08

**Authors:** Genki Kurihara, Yoshihiro Ujihara, Masanori Nakamura, Shukei Sugita

**Affiliations:** 1 Department of Electrical and Mechanical Engineering, Graduate School of Engineering, Nagoya Institute of Technology, Nagoya 466-8555, Japan; g.kurihara.251@stn.nitech.ac.jp (G.K.); ujihara.yoshihiro@nitech.ac.jp (Y.U.); nakamura.masanori@nitech.ac.jp (M.N.); 2 Center of Biomedical Physics and Information Technology, Nagoya Institute of Technology, Nagoya 466-8555, Japan; 3 Department of Nanopharmaceutical Sciences, Nagoya Institute of Technology, Nagoya 466-8555, Japan

**Keywords:** aortic dissection, delamination strength, BAPN, elastin, collagen

## Abstract

Aortic dissection (AD) is a life-threatening tear of the vascular tissue with creation of a false lumen. To explore the mechanism underlying this tissue tear, this study investigated the delamination strength of AD model rats and the histological composition of the aorta at various stages of AD development. SD rats were administrated beta-amino propionitrile for 0 (Control), 3 (Pre-dissection), and 6 (Dissection) weeks. The thoracic aorta was harvested at 10–11 weeks of age. The Dissection group exclusively showed AD at the ascending aorta. The delamination strength, a force that separates the aorta in the radial direction, of the descending aorta decreased significantly in the order of the Control, Pre-dissection, and Dissection groups. A quantitative histological analysis of the aortic tissue demonstrated that, compared with the Control group, the area fraction of collagen was significantly higher in the Pre-dissection and Dissection groups and that of elastin was significantly lower in the Dissection group. The area fraction of the elastin fibers between the elastic laminas (interlaminar fibers) was significantly decreased in the order of the Control, Pre-dissection, and Dissection groups. Histological changes of the aortic tissue, perhaps a reduction in interlaminar fibers mainly aligned in the radial direction, decreased delamination strength, thereby causing AD.

## 1. Introduction

Aortic dissection (AD) is a life-threatening disease in which the aortic wall separates in the radial direction, thus allowing blood to flow through the created lumen (false lumen) and often causing aortic rupture; the mortality rates before patients arrive at hospitals and at 24 h after rupture are reported to be 17.5% and 21.4%, respectively [1]. Furthermore, mortality rates in hospitals were 19.3–31.8% and 31.0–42.7% within one month and one year, respectively [2]. Various tissue compositional changes have been reported in AD. Elastin [3] and type IV collagen [4] is decreased and type I and III collagen are increased [5] in AD. Additionally, elastin fibers lying between elastic laminas (ELs) (interlaminar fibers) are decreased in AD [6,7]. These data suggest that the compositional changes in the aortic tissue are a major concern in the context of the occurrence and development of AD.

The delamination strength, which is the force that separates the aorta in the radial direction, can be a useful indicator in the assessment of AD risk because the aortic wall of AD separates in the radial direction. To date, the delamination strength of the porcine thoracic aorta [8,9,10], healthy human abdominal aorta [11], and the carotid artery [12] have been investigated. Pasta et al. [13] performed a delamination test of aortic aneurysms and reported a lower delamination strength compared with a normal aorta. Histologically, the aortic aneurysms exhibited decreased elastin [14,15]. Porcine thoracic aortas treated with collagenase [16] and elastase [17] showed decreased delamination strength. Taken together, these studies indicate that changes in the composition of aortic tissues seem to reduce the delamination strength, leading to the generation of AD.

Animal models of AD should prove useful to understand the mechanism underlying the occurrence of this condition. Previously, AD rat models were generated via the administration of β-aminopropionitrile (BAPN) [18,19,20], an irreversible and orally active lysyl-oxidase inhibitor, for 6–7 weeks [21,22]. The modulation of the period of administration of BAPN enabled us to prepare models at various stages of AD. Reportedly, AD models exhibit histological changes that are similar to those of natural AD; that is, decreases in interlaminar fibers [23] and increases in collagen [24].

In this study, we aimed to elucidate the mechanism of AD generation. For this purpose, we measured the composition and delamination strength of the aorta as AD gradually formed, to investigate the relationship between these two parameters.

## 2. Materials and Methods

### 2.1. Animal Model of Aortic Dissection

All animal experiments were approved by the institutional review board for animal care of the Nagoya Institute of Technology (#2022011, #2023007) and were performed according to the guidelines specified by the Guide for Animal Experimentation, Nagoya Institute of Technology. In reference to a study that observed the occurrence of AD after treatment with 0.4% BAPN for 6 weeks [21], we used three rat groups to investigate the degree of development of AD; i.e., the Control, Pre-dissection, and Dissection groups. To form the Control group, Slc: SD male rats (Japan SLC, Hamamatsu, Japan) were bred with acidic (pH 2.5–3.0) and distilled water up to 10 weeks of age. For the Pre-dissection group, 7-week-old Slc: SD rats were bred with acidic water containing 0.4% BAPN (A3134, Sigma-Aldrich, St. Louis, MO, USA) for 3 weeks. For the Dissection group, 4-week-old Slc: SD rats were bred with acidic water containing 0.4% BAPN (A3134, Sigma-Aldrich, St. Louis, MO, USA) for 6–7 weeks. Body weight and water-uptake volume were measured every 2–3 days. The water-uptake volume was determined as the difference in the volume of the water in the bottle from the previous measurement. All groups were fed with rodent diet EQ (5L37, LabDiet, St. Louis, MO, USA).

### 2.2. Tissue Preparation

The rats were sacrificed at 10–11 weeks, and descending thoracic aortas were excised as described previously [17,25,26]. Loose connective tissues were removed, and cylindrical aorta were cut along the longitudinal (*z*) direction to obtain a plate-like specimen of 3 × 6–8 mm in the *z* and circumferential (*θ*) directions. Prior to the delamination test, half of the specimen was delaminated with forceps, to separate the specimen into the intimal and adventitial sides at the media. A piece of sandpaper (#1500, Sankyo Rikagaku, Okegawa, Japan) was glued onto both sides of the delaminated part using a cyanoacrylate adhesive (#30613, Konishi, Osaka, Japan). The sandpaper was used to chuck the specimen into the delamination tester, as described below. The specimen was imaged using a digital camera (CX3, Ricoh, Tokyo, Japan). The specimen was kept in Krebs–Henseleit (KH) buffer until further experimentation.

### 2.3. Delamination Test

A T-type delamination test was conducted using a tabletop tensile tester (EZ-SX, Shimazu, Kyoto, Japan), as described previously [17]. Briefly, the sandpaper glued onto the specimen was chucked using the two gripping parts of the tester, and the upper side of the crosshead of the tester was moved upward at a speed of 0.1 mm/s. The delamination force, *T*, was measured using a load cell (SN-20N-168, Shimazu, capacity = 20 N) and recorded. The specimen was fumed with KH buffer to prevent it from drying during the test. The test was performed in air at room temperature (25 °C) within 24 h of sacrifice.

### 2.4. Histological Test

Specimens that were prepared as stated in Section 2.1 were immersed in 10% neutral-buffered saline (062-01661, Fujifilm Wako Pure Chemical, Osaka, Japan) for 24 h. The specimens were stained with either Elastica–Masson (EM) or Azan staining at the Sapporo General Pathology Laboratory (Sapporo, Japan).

The stained samples were observed using a microscope (IX73, Olympus, Tokyo, Japan) equipped with a digital microscope camera (DP73, Olympus) and a 40× (LUCPlanFLN40X) objective lens.

### 2.5. Analytical Method

All analyses described below were implemented using the ImageJ image processing software (v. 1.51i, National Institutes of Health) [17]. The functions used were present by default in Image J.

The delamination strength of the sample, *S*_d_, was obtained by fitting the following Sigmoid function:

(1) $$T_{p}\left(t\right)=\frac{S_{d}}{1+a\cdot \exp\left(-bt-c\right)},$$

to a time variation of the peeling force per unit length, *T*_p_, which was defined by:

(2) $$T_{p}\left(t\right)=\frac{T}{w},$$

where *w* is the sample width. *S*_d_, *a*, *b*, and *c* in Equation (1) are constants to be fitted. The sample width, *w*, was determined as an average of the sample widths measured at five locations.

Tissue compositions were estimated from histological images as reported previously [17]. First, we measured the area ratios of elastin, collagen, smooth muscle cells, and the background in a tissue image, in which the RGB values of each tissue component were defined as averages of 30 representative points that were clearly recognized as each component. The tunica media was then extracted as a region of interest (ROI), and the tissue component of each pixel in the ROI was determined uniquely as the one that exhibited the smallest distance to a pixel of interest in an RGB color space. After carrying out this process for all pixels, the number of pixels in each tissue component was divided by the total number of pixels in the ROI, to obtain its area fraction. EM and Azan staining images were used to determine the area fraction of elastin (*λ*_ela_) and collagen (*λ*_col_), respectively.

**Figure 1:**
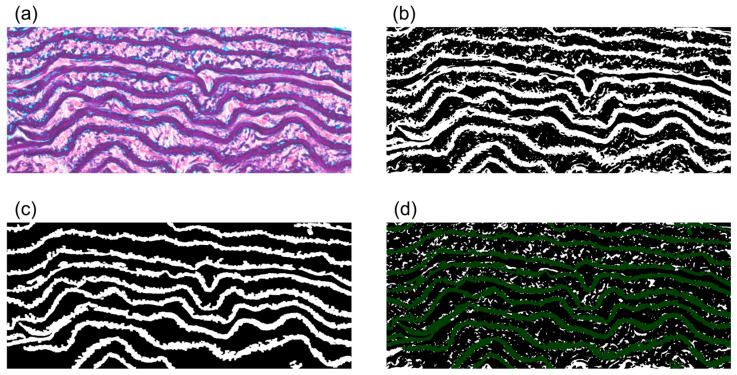
Process used to extract elastin interlaminar fibers between ELs. (**a**) Typical image of Elastica–Masson staining. (**b**) Elastin alone was extracted by thresholding the Green channel. (**c**) EL image created by selecting large and low-circularity regions in (**b**). (**d**) Image of interlaminar elastin fibers (white) obtained by subtracting panel (**c**) (dark green) from panel (**b**).

The area fraction of interlaminar fibers was calculated as schematically depicted in Figure 1. First, an elastin image was created by thresholding an EM image (Figure 1a) with the Green color, because elastin was found to have a remarkably smaller Green value versus the other components during the determination of its RGB value. Based on the elastin image (Figure 1b), small regions that were recognized as elastin were filtered out using “erode” five times, followed by “dilate” five times. An EL image mainly consisting of relatively long and thin objects was then created by selecting regions with 0.1 µm^2^ or more and a circularity of 0–0.3 using the “analyze particle” function (Figure 1c). Finally, an image of interlaminar fibers was obtained by subtracting the EL image from the elastin image using “subtract” in the “image calculator” (Figure 1d). The area fraction of interlaminar fibers, *λ*_fibers_, was calculated by dividing the area of the interlaminar fibers by that of the media.

### 2.6. Statistical Analysis

Statistical tests were performed using R software (v. 4.1.2). The delamination strength was tested via two-way repeated-measures analysis of variance (ANOVA) for the three groups of AD stage and the two directions. Data obtained between three groups were tested using the Tukey–Kramer test as the multiple comparison. The correlation coefficients, *R*, between plots were tested using Student’s *t*-test. Data are presented as raw plots and mean ± standard deviation values. The significance level was set at *p* = 0.05. Numbers of animals and data are expressed as *N* and *n*, respectively.

## 3. Results

### 3.1. Body Weight, Water-Uptake Volume, and Aortic-Dissection Generation

**Figure 2:**
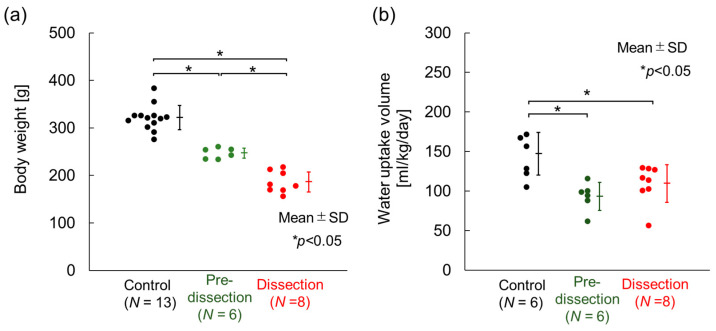
Body weight and water-uptake volume. (**a**) Body weight at sacrifice (10–11 weeks of age). (**b**) Water-uptake volume during the last week before sacrifice. *N*, number of rats. Analyzed using the Tukey–Kramer test.

Figure 2 presents the body weight of the animals at sacrifice and the daily uptake volume of water during the last week before sacrifice. The body weight was 311.7 ± 13.3 g (*N* = 13) in the Control, 247.0 ± 10.3 g (*N* = 6) in the Pre-dissection, and 186.3 ± 21.0 g (*N* = 8) in the Dissection group. Significant differences in body weight were observed among all three groups. The daily water-uptake volume was 147.1 ± 27.0 mL/kg/day (*N* = 6) in the Control, 98.9 ± 17.8 mL/kg/day (*N* = 6) in the Pre-dissection, and 113.4 ± 24.1 mL/kg/day (*N* = 8) in the Dissection group. The daily water-uptake volume of the Control group was significantly higher than that of the Pre-dissection and Dissection groups. These data were consistent with previous reports showing that the weight [18,21,24] and water-uptake volume [21] were decreased after BAPN administration. All experimental data, including the following sections, can be obtained from the Supplementary Materials (Raw data).

**Figure 3:**
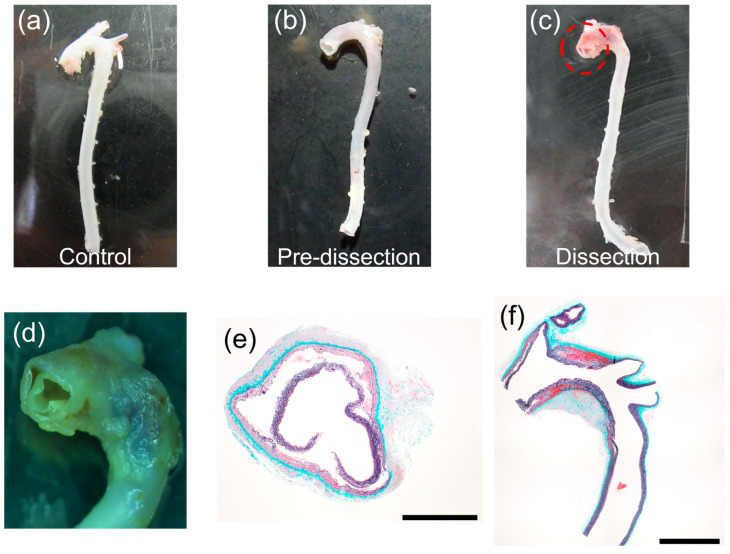
Typical images of the obtained thoracic aortas. (**a**–**c**) Whole images of the thoracic aortas of (**a**) the Control, (**b**) the Pre-dissection, and (**c**) the Dissection groups. (**d**) Magnified image of the ascending thoracic aorta (red circle in (**c**)). (**e**,**f**) Elastica–Masson staining images of the Dissection group in (**e**) axial and (**f**) sagittal cross-sections of AD regions. Bars in (**e**) = 1 mm and (**f**) = 2 mm.

Figure 3a–c provides example images of the excised thoracic aorta of the three groups. The aorta was intact in the Control and Pre-dissection groups, whereas AD was observed exclusively in the Dissection group, in which the ascending thoracic aorta was torn (Figure 3c,d). The cross-sectional image of the Elastica–Masson staining of the AD region clearly showed separation of the media at the ascending thoracic aorta and the aortic arch (Figure 3e,f). In all groups, no AD was found in the descending aortas in which the delamination test was performed.

### 3.2. Delamination Strength

**Figure 4:**
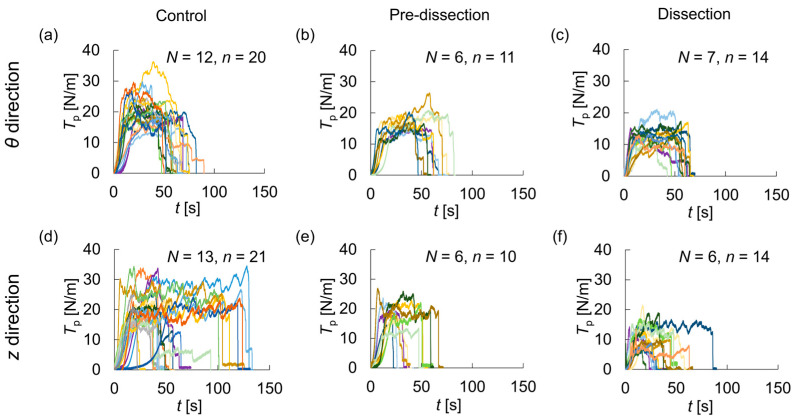
Peel tension, *T*_p_, with time, *t*. Delamination test in the (**a**–**c**) *θ*-direction and (**d**–**f**) *z*-direction in the (**a**,**d**) Control, (**b**,**e**) Pre-dissection, and (**c**,**f**) Dissection groups.

The time variation of the peel tension per unit length is shown in Figure 4 and Supplementary Videos S1–S6. Overall, the peel tension, *T*_p_, increased up to about 10 s, remained almost constant for about 50–100 s, and dropped suddenly by failure. Each specimen required a different amount of time for specimen failure. In each group, the *z*-direction delamination test (Figure 4d–f) of a part of the specimens ended in a shorter time than did the *θ*-direction delamination test (Figure 4a–c). In these cases, specimens failed because the delaminated part deviated to either the adventitial or intimal side (Video S5).

**Figure 5:**
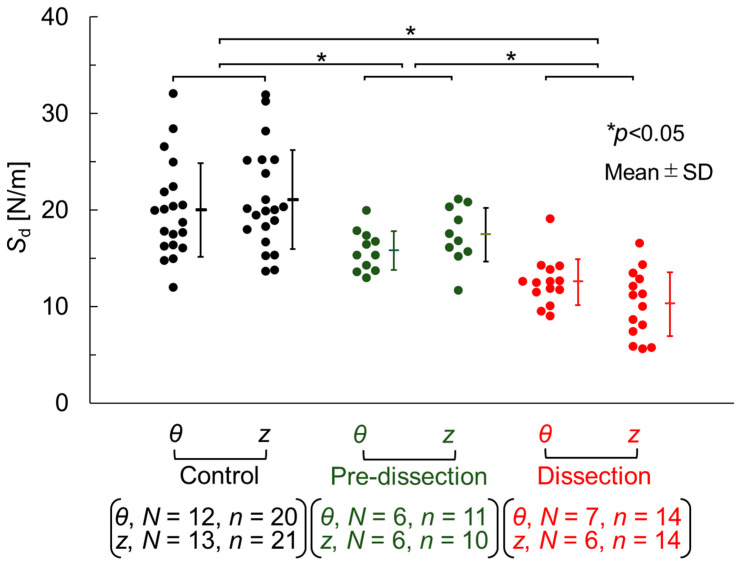
Delamination strength, *S*_d_, in the three groups. *N*, number of rats; *n*, number of samples. Tested by two-way repeated-measures ANOVA, followed by the Tukey–Kramer test.

Figure 5 depicts the delamination strength of the three groups. In the Control group, the delamination strength was 20.0 ± 4.9 N/m (*n* = 20) in the *θ*-direction and 21.1 ± 5.1 N/m (*n* = 21) in the *z*-direction. In the Pre-dissection group, the delamination strength was 15.8 ± 2.0 N/m (*n* = 11) in the *θ*-direction and 17.4 ± 2.8 N/m (*n* = 10) in the *z*-direction. In the Dissection group, the delamination strength was 12.5 ± 2.4 N/m (*n* = 14) in the *θ*-direction and 10.2 ± 3.3 N/m (*n* = 14) in the *z*-direction. A two-way repeated-measures ANOVA revealed significant differences only at the AD stages. Post-hoc testing identified significant differences among all three groups. These statistical examinations demonstrated that the delamination strength decreased in the order of the Control, Pre-dissection, and Dissection groups. Although significant differences were not observed between the *z*- and *θ*-directions, the average delamination strength in the *z*-direction appeared to be larger than that detected in the *θ*-direction in the Control and Pre-dissection groups, and smaller in the Dissection group.

### 3.3. Histological Analysis

**Figure 6:**
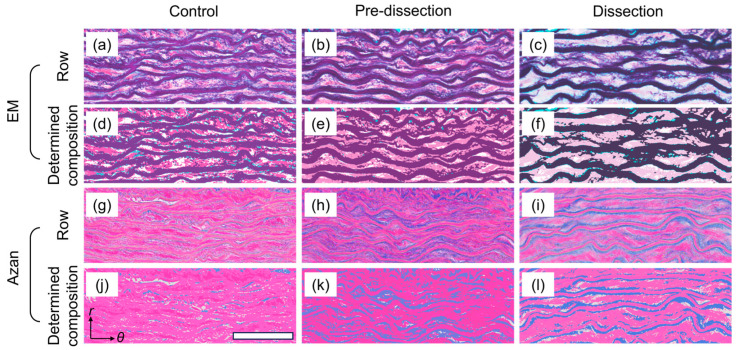
Histological images and determination of their composition. (**a**–**f**) Typical images of (**a**–**c**) Elastica–Masson (EM) staining and (**d**–**f**) corresponding images that were reconstructed by identifying the tissue composition for each pixel in the (**a**,**d**) Control, (**b**,**e**) Pre-dissection, and (**c**,**f**) Dissection groups. Elastin, collagen, smooth muscle cells, and the background are shown in dark purple, light blue, pink, and white, respectively. (**g**–**l**) Typical images of (**g**–**i**) Azan staining and (**j**–**l**) the composition determined in the (**g**,**j**) Control, (**h**,**k**) Pre-dissection, and (**i**,**l**) Dissection groups. Collagen, elastin, smooth muscle cells, and the background are shown in blue, pink, red, and white, respectively. Bar in (**j**) = 50 μm. *r*, radial; and *θ*, circumferential directions.

Figure 6 provides typical histological images. Elastica–Masson staining images revealed a decrease in elastin fibers, especially between ELs in the Dissection group (Figure 6c), compared with those in the Control (Figure 6a) and Pre-dissection (Figure 6b) groups. The ELs in the Dissection group (Figure 6c) appeared thicker than those in the Control (Figure 6a) and Pre-dissection (Figure 6b) groups. The collagen amount seemed to be increased in the Pre-dissection (Figure 6h) and Dissection (Figure 6i) groups compared with the Control group (Figure 6g), especially near ELs. Images that were reconstructed by assigning one of the tissue compositions to each pixel (Figure 6d–f,j–l) were similar to the original images (Figure 6a–c,g–i), thus supporting the validity of digital identification of tissue compositions.

**Figure 7:**
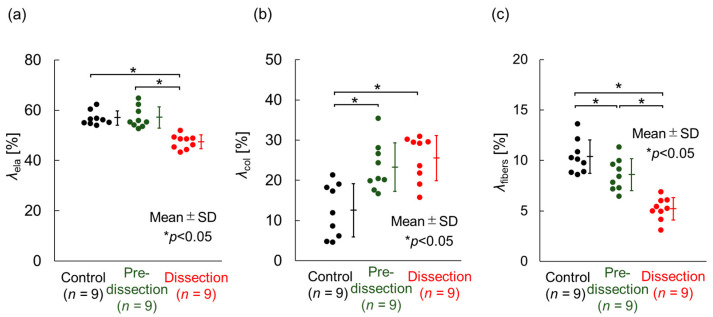
Composition of the thoracic aorta at three aortic-dissection stages. (**a**) The area fraction of elastin, *λ*_ela_, in Elastica–Masson staining. (**b**) Area fraction of collagen, *λ*_col_, in Azan staining. (**c**) Area fraction of interlaminar fibers between ELs *λ*_fiber_. *n*, number of data. Analyzed using the Tukey–Kramer test.

Figure 7a reports the area fraction of elastin. The area fractions of elastin were 56.9 ± 2.8% (*n* = 9) in the Control, 57.1 ± 4.2% (*n* = 9) in the Pre-dissection, and 47.2 ± 2.8% (*n* = 9) in the Dissection group. The area fraction of elastin in the Dissection group was significantly lower than area fractions in the Control and Pre-dissection groups. This result indicates that the elastin density decreased at the Dissection stage.

Figure 7b depicts the area fraction of collagen. The area fractions of collagen were 12.5 ± 6.7% s (*n* = 9) in the Control, 23.3 ± 6.0% (*n* = 9) in the Pre-dissection, and 25.5 ± 5.6% (*n* = 9) in the Dissection groups. The area fraction of collagen in the Control group was significantly lower than that in the Pre-dissection and Dissection groups. This result indicates that the collagen density increased at the Pre-dissection stage.

Figure 7c presents the area fraction of interlaminar fibers. The area fractions of interlaminar fibers were 10.4 ± 1.6% (*n* = 9) in the Control, 8.6 ± 1.6% (*n* = 9) in the Pre-dissection, and 5.2 ± 1.2% (*n* = 9) in the Dissection groups. Significant differences were detected among all three groups. This result indicates that interlaminar fibers decreased gradually as the AD developed.

**Figure 8:**
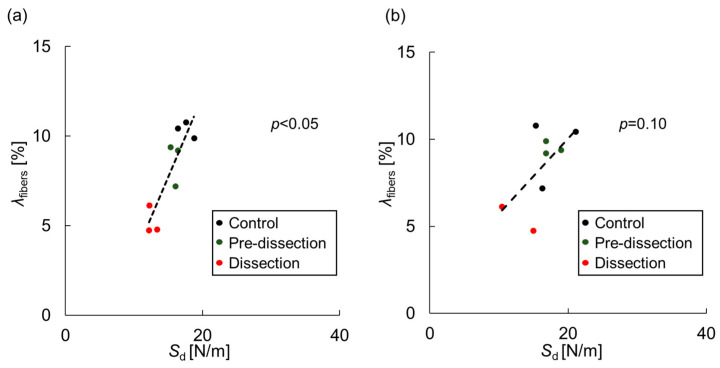
Relationship between the delamination strength, *S*_d_, and the area fraction of interlamellar fibers, *λ*_fibers_, in the (**a**) *θ*-direction and (**b**) *z*-direction. Analyzed by *t*-tests.

Figure 8 shows the relationship between the delamination strength and the area fraction of interlaminar fibers, obtained from specimens on which both tests were performed. Although the correlation was insignificant in the *z*-direction delamination test (*p* = 0.10), the delamination strength correlated significantly with the area fraction of interlaminar fibers in the *θ*-direction delamination test. This result indicates that the reduction of interlamellar fibers decreases the delamination strength.

## 4. Discussion

We performed a delamination test on AD model rats and histologically investigated the aortic tissue at various stages of AD development. The main findings were as follows: as AD developed, (1) the delamination strength decreased, (2) the area fraction of interlaminar fibers decreased, and (3) the area fraction of collagen increased first, followed by a decrease in that of elastin. These results are novel, to the best of our knowledge.

A reduction in the delamination strength is the likely cause of AD. The delamination strength decreased in the order of the Control, Pre-dissection, and Dissection groups, as shown in Figure 5. AD did not occur in the Pre-dissection group, although its delamination strength was lower than that of the Control group. A further decrease in delamination strength resulted in a tear of the ascending aorta, or AD, in the Dissection group, suggesting that the aortic tissue could not bear the delamination force.

The area fraction of interlaminar fibers decreased in the order of the Control, Pre-dissection, and Dissection groups. This observation was congruent with the report of Nakashima et al. [23], who showed a significant reduction in interlaminar elastic fibers in rats treated with BAPN. Reportedly, BAPN binds to vitamin B6, which is required for the crosslinking of elastin as a co-enzyme by lysyl-oxidase [27]. The administration of BAPN inhibits lysyl-oxidase activity [28] and, thus, the crosslinking of elastin. These findings suggest that the reduction of interlaminar fibers observed in the present study was caused by the decrease in elastin crosslinking resulting from BAPN administration. A reduction of interlaminar fibers was also detected in human AD [6]; thus, the triggering of AD is attributable to the reduction of interlaminar fibers.

Both the interlaminar fibers and delamination strength decreased with the development of AD. The interlaminar fibers are mainly aligned in the *r* direction [29], and a part of them crosslinks with the adjacent ELs. Thus, it is reasonable to consider that interlaminar fibers normally resist the force to separate the aorta in the *r* direction. In that case, the reduction of interlaminar fibers decreases the delamination strength in the *r* direction. Based on these findings, we hypothesized that the decrease in the interlaminar fibers caused a reduction in the delamination strength, and accordingly, the aortic wall could not bear the radial force possibly brought by high blood pressure, leading to the tear of the aorta.

The decrease in delamination strength that accompanies AD development would also be attributable to changes in other tissue components, i.e., collagen and elastin. As shown in Figure 6b, the area fraction of collagen in the Pre-dissection group was larger than that in the Control group. Reportedly, the administration of BAPN increased the collagen ratio and disrupted the crosslinks between collagen fibrils and fibers [24]. The same phenomenon would have occurred in the Pre-dissection group; the disruption of the crosslinks by BAPN administration may have reduced the tensile strength of the collagen fibers that aligned in the *r* direction of the aorta, thus decreasing the delamination strength. Further reduction of the delamination strength would have been promoted in the Dissection group (Figure 5). Because elastin is a key determinant of delamination strength [17], the decreased delamination strength observed between the Pre-dissection and Dissection groups may have been induced by the elastin reduction that occurred after the collagen increase.

**Figure 9:**
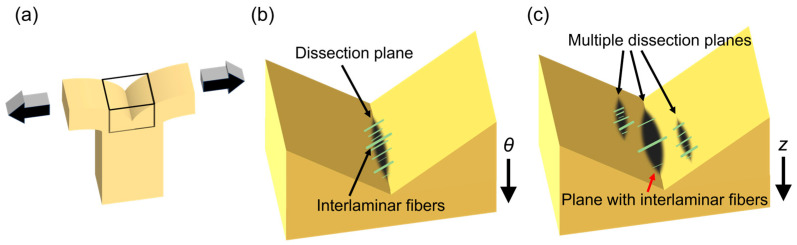
Possible mechanism underlying the more proactive decrease in *z*-delamination strength observed after the administration of BAPN. (**a**) Specimen during the delamination test. (**b**,**c**) Magnified views of the boxed area. (**b**) Specimen in the *θ*-direction delamination test showing a single dissection plane. (**c**) Specimen in the *z*-direction delamination test showing multiple dissection planes with different numbers of interlamellar fibers.

The average delamination strength determined by the *z*-directional delamination test (*z*-delamination strength) was higher than that recorded by the *θ*-directional test (*θ*-delamination strength) in the Control and Pre-dissection groups (Figure 5), similar to that reported previously for human abdominal aortas [11] and carotid arteries [12]. Further reduction in the *z*-delamination strength inverted the order of the delamination strength in the Dissection group; the *θ*-delamination strength became larger than the *z*-delamination strength. The reduction of the *z*-delamination strength may have been caused by a combination of two factors; firstly, the loss of interlamellar elastin fibers. In a previous study [17], we performed a delamination test of the normal aorta and proposed a possible mechanism accounting for the higher delamination strength observed in the *z* direction versus the *θ* direction. Briefly, interlamellar fibers mainly align in the *r* direction and are slightly tilted in the *θ* direction. Such tilting of the fibers provides greater *z*-delamination strength compared with *θ*-delamination strength, as observed in the Control group. In the Dissection group, interlamellar fibers were decreased because of the administration of BAPN. This would have reduced *z*-delamination strength, rendering it lesser than the *θ*-delamination strength. The second factor is the generation of multiple dissection planes in the delamination test used to determine *z*-delamination strength. Figure 9 illustrates the dissection planes in the delamination test of (b) *θ*-direction delamination and (c) *z*-direction delamination strength. Yu et al. [30] carried out a similar delamination test using normal aortas. The authors reported that the dissection plane became multiple in the *z*-direction delamination test (Figure 9c), whereas it remained single in the *θ*-direction delamination test, for unknown reasons. Aortic dissection progresses preferentially from a weaker part of the tissue. Because a sample with multiple dissection planes has more chances of having a dissection plane with fewer interlamellar fibers, delamination occurs more easily in the sample with multiple dissection planes; thus, this sample has weaker delamination strength. This effect would have been pronounced in the Dissection group, in which the interlamellar fibers were markedly reduced by BAPN administration. This combination of two factors resulted in the lowering of *z*-direction delamination strength compared with *θ*-direction delamination strength in the Dissection group.

The time to complete the delamination test differed between specimens, which was simply explained by the different lengths of the specimens in the delamination direction. Interestingly, the completion time in the *z*-direction delamination test was shorter in some specimens compared with that in the *θ*-direction delamination test. One explanation for this observation would be the lateral deviation of the dissection plane caused by the administration of BAPN. Sommer et al. [11] reported that the dissection plane crossed the ELs in the z-direction delamination test of the normal aorta. This phenomenon could have also occurred in the present study. In fact, we observed such a phenomenon in our delamination test, as shown in Video S5. The reduction of elastin fibers caused by BAPN administration might have made the dissection reach the intimal or adventitial side in the Pre-dissection and Dissection groups.

There are some limitations to this study. To begin, data obtained from the rat AD model might not be true of the human AD. However, a decrease in interlamellar fibers was observed in both the rat AD model and human AD, indicating that the rat AD model used in this study has similar properties to human AD. Moreover, there have been no studies investigating the delamination strength of rat aorta, to our best knowledge; thus, we cannot compare these results to previous data. The delamination strengths of porcine specimens were 53.0 ± 17.6 N/m in the *θ*-direction and 71.4 ± 32.1 N/m in the *z*-direction [17], which are higher than the rat specimens obtained in this study. Since the delamination strength of the human aorta [13] was also greater than the one in this study, we cannot eliminate the probability of differences between rats and humans in the structure of aortas.

## 5. Conclusions

The delamination strength of thoracic aortas was significantly lower after the administration of BAPN compared with control cases. The area fraction of elastin fibers between the ELs decreased with the development of AD. BAPN administration first increased the area fraction of collagen, followed by a decrease in area fraction of elastin. These histological changes in the aorta are the likely cause of the decrease in its delamination strength, thereby triggering AD.

## Data Availability

Data are contained within the article.

## Supplementary Materials

The following supporting information can be downloaded at: https://www.mdpi.com/article/10.3390/bioengineering10111292/s1, Video S1: Circumferential delamination test in the Control group. Video S2: Circumferential delamination test in the Pre-dissection group. Video S3: Circumferential delamination test in the Dissection group. Video S4: Longitudinal delamination test in the Control group. Video S5: Longitudinal delamination test in the Pre-dissection group. Video S6: Longitudinal delamination test in the Dissection group. Raw data: Data of this study.
